# Recent Advances in Polymeric Materials Used as Electron Mediators and Immobilizing Matrices in Developing Enzyme Electrodes

**DOI:** 10.3390/s120100923

**Published:** 2012-01-16

**Authors:** Mambo Moyo, Jonathan O. Okonkwo, Nana M. Agyei

**Affiliations:** 1 Department of Environmental, Water, and Earth Sciences, Tshwane University of Technology, Private Bag X680, Arcadia, Pretoria 0001, South Africa; E-Mail: OkonkwoOJ@tut.ac.za; 2 Department of Chemistry, University of Limpopo, P.O. Box 235, Medunsa 0204, South Africa; E-Mail: agyei@ul.ac.za

**Keywords:** polymeric material, mediator, electrochemical sensor, biosensor, enzyme electrode

## Abstract

Different classes of polymeric materials such as nanomaterials, sol-gel materials, conducting polymers, functional polymers and biomaterials have been used in the design of sensors and biosensors. Various methods have been used, for example from direct adsorption, covalent bonding, crossing-linking with glutaraldehyde on composites to mixing the enzymes or use of functionalized beads for the design of sensors and biosensors using these polymeric materials in recent years. It is widely acknowledged that analytical sensing at electrodes modified with polymeric materials results in low detection limits, high sensitivities, lower applied potential, good stability, efficient electron transfer and easier immobilization of enzymes on electrodes such that sensing and biosensing of environmental pollutants is made easier. However, there are a number of challenges to be addressed in order to fulfill the applications of polymeric based polymers such as cost and shortening the long laboratory synthetic pathways involved in sensor preparation. Furthermore, the toxicological effects on flora and fauna of some of these polymeric materials have not been well studied. Given these disadvantages, efforts are now geared towards introducing low cost biomaterials that can serve as alternatives for the development of novel electrochemical sensors and biosensors. This review highlights recent contributions in the development of the electrochemical sensors and biosensors based on different polymeric material. The synergistic action of some of these polymeric materials and nanocomposites imposed when combined on electrode during sensing is discussed.

## 1. Introduction

**Figure 1.**
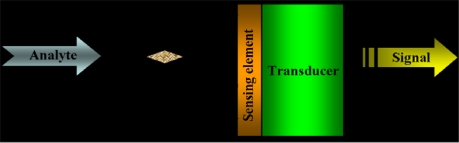
Schematic diagram of a biosensor device.

Classical enzyme electrodes are obtained by combining directly the enzyme and surface of electrodes of platinum, gold, carbon paste or glassy carbon. Electrical conductivity and the hardness of the material are the major factors to be considered when selecting electrode material [1]. The enzyme can be combined with the electrode through covalent bonding on the electrode, cross-linking with the polymer or physical absorption. The development of amperometric, potentiometric or conductometric enzyme electrodes is to design the interface between the transducer and the biocatalytical layer so as to allow efficient electron transfer between enzyme and the electrode. In these devices, the function of the enzyme is to provide selectivity by virtue of its biological affinity for a particular substrate molecule that is of biological origin [1]. In this case a biosensor (Figure 1) is formed and can be defined as a “self-contained integrated device that is capable of providing specific quantitative or semi-quantitative analytical information using a biological recognition element (biochemical sensing element) which is in direct spatial contact with a transduction element [2].

These biosensors have recently attracted much interest because they have been shown to have applications in clinical diagnostics, environmental monitoring, food freshness and bioprocess monitoring [3]. The performance of an enzyme electrode is largely governed by the materials used. Enzymatically generated redox reactions are transferred by means of freely diffusing natural or artificial redox mediators via a shuttle mechanism [4]. Husain and Husain defined redox mediators as the compounds that speed up the reaction rate by shuttling electrons from the biological oxidation of primary electron donors or from bulk electron donors to the electron-accepting organic compounds [5]. An appropriate working potential is applied to the modified electrode leading to oxidation or reduction of mediators at electrode surface. Current is generated which is proportional to the concentration of substrate. Oxidation or reduction of electroactive species on the surface of modified electrodes are widely used to evaluate its activity further to develop biosensors. However, the direct oxidation of electroactive specie such as thiocholine at classical electrodes is slow, prone to interference problems from other electroactive species and also requires high overpotential with the added problem of electrode fouling [6–8]. Schuhmann reported that free-diffusing redox mediators cannot be securely retained at the biosensor surface and their leakage leads to a limited long-term stability of the electrode [4]. In this communication, the main emphasis is on discussion of the different classes of polymeric materials that have been investigated as to their ability to overcome these drawbacks and to enable amperometric detection at low potentials of without passivation. This will also allow a possible move from the application of these expensive polymeric materials to low cost materials or blending of these low cost biomaterials so that they can be used as electron mediators.

## 2. Types of Materials Used as Electron Mediators and Immobilizing Matrices

This section focuses on the different classes of polymeric materials namely nanomaterials, sol-gel materials, conductive polymeric materials, functional polymeric materials and biomaterials that have been used as electron mediators and immobilizing matrix in the design of sensors and biosensors. The applicability of each of these polymeric materials is discussed in each section.

### 2.1. Nanomaterials

#### 2.1.1. Carbon Nanotubes

**Figure 2.**
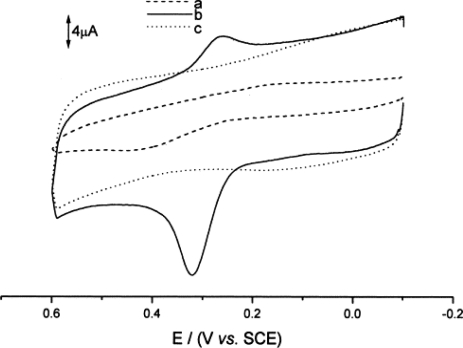
Cyclic voltammograms at a bare GC electrode (**a**) and a SWNT film-modified GC electrode (**b**,**c**) in the absence of DOPAC (c) and in the presence of 6.0 × 10^−5^ M DOPAC (a,b) in 0.1 M HAc-NaAC buffer solution (pH 4.4). Scan rate 0.1 V s^−1^. Reprinted with permission from [17].

The accuracy, efficiency and detection limit of a biosensor can be increased with the use of carbon nanotubes immobilized on bare electrodes. Carbon nanotubes [9], due to their unique electrochemical and mechanical properties (high electron transfer, high surface area, minimization of the surface fouling, high stability, excellent adsorptive and biocompatibility), have been successfully used for fabricating electrochemical sensors and biosensors [10–16]. The electrocatalytic oxidation of 3,4-dihydroxyphenylacetic acid (DOPAC) at a glassy carbon electrode (GCE) modified with single-walled carbon nanotubes was used to illustrate the unique properties of the nanotubes in a potential range of −0.1 V to +0.6 V (*vs.* SCE) [17]. The electrochemical behaviour of DOPAC at this SWNT-modified electrode was shown to be a diffusion controlled process. The peak current increased linearly with concentration of DOPAC in the range of 1.0 × 10^−6^–1.2 × 10^−4^ M and a detection limit of 4.0 × 10^−7^ M was obtained. The above characteristics demonstrated that the modified electrode might be used in biosensors to study electrochemistry of biomolecules since carbon nanotubes provided good electrocatalytic properties as mediators compared to a bare electrode (Figure 2).

In another study, a comparison of the electrochemical and electroanalytical behaviour of ascorbic acid, dopamine and uric acid at bare, activated and multi-wall carbon nanotubes modified glassy carbon electrodes was demonstrated by Zare and Nasirizadeh [18]. The potential ranges for the bare GCE (−0.3 to +0.9 V), activated GCE (+0.04 to +0.76 V) and MWCNT-GCE (+0.1 to +0.79 V) were used. The MWCNT-GCE was quite effective at this low potential range not only for oxidation of the three acids separately but also in the simultaneous determination of each component in the mixtures.

#### 2.1.2. Carbon Nanotube Composites

Composite films of carbon nanotubes with other materials such as enzymes, conducting polymers *etc*. are very attractive combinations of material for the development of electrochemical sensors and biosensors. Li *et al*. reported the formation of covalently linked composites of multi-walled carbon nanotubes (MWCNT) and glucose oxidase (GO_x_) for glucose biosensing [19]. The electrochemical characterization of the GO_x_-MWCNT composites on glassy carbon electrode by atomic force microscopy (AFM) and transmission electron microscopy (TEM) showed that the covalently linked GO_x_ retained its bioactivity and could specifically catalyse the oxidation of glucose. The oxidation current, anodic peak current (ip_a_), from cyclic voltammetry showed a linear dependence on glucose concentration in the solution in the range of 0.5–40 nM with detection limit of 30 μM and a detection sensitivity of 11.3 μA/mM cm^2^. Deo *et al.* demonstrated the use of carbon nanotubes (CNTs) for enhancing the amperometric biosensing of organophosphate pesticides [20]. A bilayer approach with organophoshorus (OPH) layer atop of the CNT film was used for preparing the CNT/OPH biosensor. The CNT layer led to a greatly improved anodic detection of the enzymatically generated *p*-nitrophenol product, higher sensitivity and stability. The biosensor was used to measure as low as 0.15 μM paraoxon and 0.8 μM methyl parathion with sensitivities of 25 and 6 nA/μM, respectively.

To illustrate the synergistic effect of various polymeric materials, the MWCNT were used as an immobilization matrix to incorporate thionin as electron transfer mediator onto a GCE surface [21]. Due to unique electronic properties of MWCNTs and electrocatalytic activity of thionin, the combination of thionin and MWCNTs resulted in a remarkable synergistic augmentation on the response. Chitosan (CS), a biological cationic macromolecule, provides a suitable environment for enzymes to retain their specific activity. Their combination with nanotubes to form a film for GCE has stimulated much interest.

**Figure 3.**
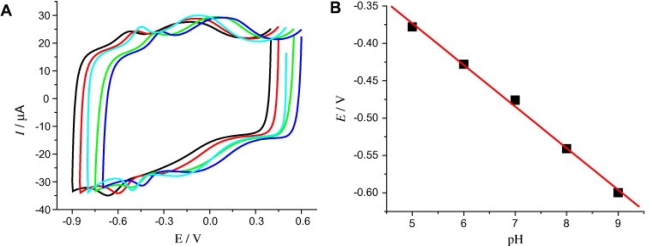
(**A**) CVs of catalase modified GCE in different pH solutions: from right to left, 5, 6, 7, 8, and 9 (scan rate 0.1 V s^−1^); (**B**) Plot of formal potential *versus* pH values. Reprinted with permission from [22].

Jiang *et al*. reported on the electrochemical performance of catalase (CAT) immobilized on a film composed of chitosan-wrapped single-walled carbon nanotubes (SWNTs), and the use of such a system as a biosensor [22]. A pair of well-defined and quasi-reversible redox peaks, with a formal potential (E*˚^1^*) of −0.476 V *vs*. SCE in 0.1 M, pH 7.0 phosphate buffer solution was obtained. The dependence of formal potential (values obtained from cyclic voltammograms, Figure 3(A)) on solution pH as illustrated in Figure 3(B) shows that that the direct electron transfer reaction of CAT is a one-electron transfer coupled with a one-proton transfer reaction. The heterogeneous electron transfer rate constant was measured as 118 s^−1^, indicating electron transfer between catalase and the modified electrode surface is greatly improved over that which is typically reported in the literature. This result was attributed to the close contacts between the electroactive centers and the SWNTs. Experimental results demonstrated that absorbed CAT exhibited remarkable electrocatalytic activities toward the reduction of oxygen, hydrogen peroxide and nitrite. Yuan *et al.* reported on Streptavidin being used as a molecular linker to immobilize biotinylated acetylcholinesterase (AChE) on carbon nanotubes (CNTs) in a gentle and controllable fashion for pesticide biosensors [23]. Glassy carbon electrodes coated with the CNT-enzyme complex had high affinity for the substrate acetylthiocholine and produced strong peak oxidation currents in electrochemical assays.

The construction of a robust and selective galactose biosensor by careful optimization of biosensor composition and physicochemical properties was demonstrated by Tkac *et al.* [24]. In this study, chitosan was chosen as a natural polymer for dispersion of SWNT based on its ability to efficiently solubilize nanotubes to form a stable dispersion. Stabilisation of the chitosan film containing single-walled nanotubes (CHIT-SWNT) was done by chemical cross-linking with glutaraldehyde and free aldehyde groups produced a substrate used for covalent immobilization of galactose oxidase (GalOD). A novel method for immobilization of acetylcholinesterase (AChE) to cross-linked chitosan-MWNTs composite through covalent bonding was also reported by Ju and Kandimalla [25]. Zheng *et al.* modified a glassy carbon electrode (GC) using multi-walled carbon nanotube (MWCNTs) [26]. The modified electrode showed a pair of redox peaks that resulted from the oxygen-containing functional groups on the nanotubes surface as evidenced by FTIR spectrum during their study. A recombinant thermostable dye-linked l-proline dehydrogenase (l-proDH) from hyperthermophilic archaeon (*Thermococcus profundus*) was further immobilized by physical adsorption. The GC/MWCNTs/l-proDH electrode exhibited an electrocatalytical signal for l-proline compared to bare GC, GC/l-proDH and GC/MWCNTs electrodes, which suggested that the presence of MWCNTs efficiently enhances electron transfer between the active site of enzyme and electrode surface. A typical Michaelis-Menten catalytic response with lower apparent constant was reported.

Kumar *et al.* reported on an amperometric biosensor fabricated for the detection of carbaryl based on single-walled carbon nanotubes (SWCNTs) and AChE [27]. The dispersion of SWCNTs in positively charged polyelectrolyte, poly(diallydimethylammonium chloride) possibly occurred due to weak supramolecular interaction. Qian and Yang designed a new amperometric biosensor for hydrogen peroxide based on cross-linking horseradish peroxidase (HRP) by glutaraldehyde with multiwall carbon nanotubes/chitosan (MWNTs/CH) composite film coated on a glassy carbon electrode [28]. MWNTs were firstly dissolved in a chitosan solution and the morphology of MWNTs/CH composite film was characterized by field-emission scanning electron microscopy. MWNTs were well soluble in chitosan and robust films could be formed on the surface as evidenced from the SEM picture. HRP was cross-linked by glutaraldehyde with MWNTs/CH film to prepare a hydrogen peroxide biosensor. The enzyme electrode exhibited excellent electrocatalytic activity and rapid response for H_2_O_2_. The linear range of detection was from 1.67 × 10^−5^ to 7.40 × 10^−4^ M with correction coefficient of 0.998. The biosensor had good repeatability and stability for the determination of H_2_O_2_ and interferences problems from other compounds were investigated. Acetylcholinesterase biosensor based on carbon nanotube paste in the determination of chlorphenvinphos was evaluated by Oliveira and Mascaro [29]. Using a chronoamperometric procedure, a linear analytical curve was observed in the range 4.90 × 10^−7^–7.46 × 10^−6^ M, with a limit of detection of 1.15 × 10^−7^ M. The determination of this insecticide proved to be in agreement with the standard spectrophotometric method, with a 95% confidence level and a relative error lower than 3%.

Du *et al.* presented a simple and efficient method for immobilization of AChE on a MWNT cross-linked chitosan composite (CMC)-modified glassy carbon electrode [30]. The constructed matrix used glutaraldehyde as cross-linker preventing leakage of enzyme from the film. Chitosan provided a biocompatible microenvironment around the enzyme and maintained its high biological activity. The immobilized enzyme had greater affinity for acetylthiocholine and excellent catalytic effect with a K_m_^app^ value of 132 μmol/L, forming thiocholine, which was oxidized to produce detectable and rapid response. Du and Cai reported on the enzyme acetylcholinesterase (AChE) which was immobilized through covalent bonding to a MWNT cross-linked cellulose acetate composite on a screen-printed carbon electrode (SPCE) [31]. The immobilized enzyme was preserved on this film because of the excellent biocompatibility and non-toxicity of cellulose acetate. The reported AChE biosensor had greater affinity for acetylthiocholine (ATCl) and excellent catalytic effect in the hydrolysis of ATCl in a potential range of 0.0 to 1.0 V. Carbaryl in garlic samples was successfully determined using this biosensor through enzyme inhibition. The detection limit was 0.04 μg/mL and was taken as the concentration equivalent to 10% decrease in signal.

Xie *et al.* chose Nafion for dispersion of insoluble MWNTs in ethanol due to its surface wrapping effect, resulting in a stable, well distributed MWNTs/Nafion film suspension [32]. Moreover, the MWNTs/Nafion film-modified glassy carbon electrode exhibits catalytic ability to electrochemical reduction of Pb^2+^ due to large cation exchange capacity, strong adsorption ability, large surface area and subtle electronic property of the composite. In another work, Tiwari prepared a nanocomposite made up of silylated chitosan and multiwall carbon nanotubes (CHIT-SiO_2_-MWCNTs) for immobilization of urease (Urs) [33]. Urs enzyme was covalently immobilized with the CHIT-SiO_2_-MWCNTs matrix using glutaraldehyde as a linker. The relatively low Michaelis-Menten constant of 0.15 mM indicated that the CHIT-SiO_2_-MWCNTs matrix had a high affinity for the Urs enzyme and demonstrated the excellent electrocatalytic activity of MWCNTs. Wei *et al*. reported on the electrochemical impedance determination of polychlorinated biphenyl using a pyrenecyclodextrin decorated single-walled carbon nanotube hybrid [34]. Recently, a highly sensitivity modified electrode compared to the unmodified one was demonstrated by Kuralay and coworkers [35], who used a single walled-chitosan composite to modify a desposable pencil graphite electrode (PGE). The modified electrode was further used in the electrochemical detection of Vitamin B_12_. The modified electrode showed high catalytic activity and high selectivity due to the presence of nanotubes as compared to bare PGE. Fast electron transfer coupled with high stability was also observed. Yin *et al.* invented an amperometric bisphenol A (BPA) biosensor fabricated by immobilizing tyrosinase on multi-walled carbon nanotubes (MWNTs)-cobalt phthalocyanine (CoPc)-silk fibroin (SF) composite modified GCE [36]. In MWNTs-CoPc-SF composite film, SF provided a biocompatible microenvironment for the tyrosinase to retain its bioactivity, MWNTs possessed excellent inherent conductivity to enhance the electron transfer rate and CoPc showed good electrocatalytic activity to electrooxidation of BPA. A well-defined anodic peak was observed at 0.625 V and compared with bare GCE, the oxidation signal of BPG was increased.

#### 2.1.3. Metal Nanoparticles

The uses of metal nanoparticles in electrochemical sensing have received considerable attention in recent years. Incorporation of nanoparticles to CNTs for the modification of electrodes has been demonstrated to enhance the electrocatalytic activity of many electrochemical processes and, therefore, be suitable for sensing and biosensing applications [15]. Direct electrochemistry of glucose oxidase immobilized on chitosan-gold nanoparticle composite film on a glassy carbon electrodes and its biosensing application was reported by Sheng *et al.* [37]. The characteristic factor such as electron transfer rate constant was estimated to be 15.6 s^−1^ and this indicated the presence of high electron transfer between the GO_x_ redox center and the electrode. The presence of both chitosan and gold nanoparticle enhanced the stability of the GO_x_ in the composite film and retained its bioactivity. The calculated apparent Michaelis-Menten constant was 10.1 mmol/L. Tan *et al.* reported on novel magnetic Fe_3_O_4_/chitosan (CS) microspheres which were prepared using magnetic Fe_3_O_4_ nanoparticles and the natural macromolecule chitosan [38]. Using a hemoglobin (Hb) immobilization method, an innovative biosensor with a Fe_3_O_4_/CS-Hb-Fe_3_O_4_/CS “sandwich” configuration was constructed. This biosensor had a fast (less than 10 s) response to H_2_O_2_ and excellent linear relationships were obtained in the concentration range of 5.0 × 10^−5^ to 1.8 × 10^−3^ M and 1.8 × 10^−3^ to 6.8 × 10^−3^ M with a detection limit of 4.0 × 10^−6^ M. The apparent Michaelis-Menten constant was 0.29 mM and this displayed the excellent biological activity of the fixed Hb. Furthermore, the biosensor experienced long-time stability and good reproducibility. Huang *et al.* developed a biosensor with superior accuracy and sensitivity based on an interdigital transducer (IDA) microelectrode [39]. The IDA microelectrode was prepared by a photolithography procedure with sputter coated thin layers of gold and SiO_2_, followed by tetrafluoromethane (CF_4_) plasma treatment. The enzymatic interactions between glucose and the reactants were monitored by cyclic voltammetry. The developed IDA-microelectrode-based glucose biosensor showed particular precision and excellent reproducibility in measuring glucose concentration under the presence of common interferences and in real human blood measurements. Moreover, the IDA microelectrodes displayed a sensitivity of 0.13 μA/(mg/dL) which is much higher than commercially available products.

**Figure 4.**
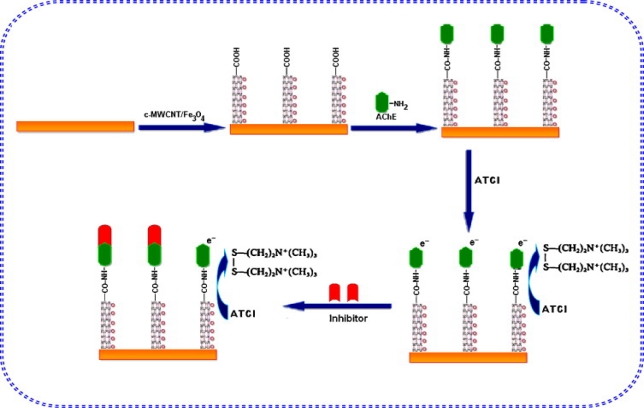
Scheme for the stepwise amperometric biosensor fabrication process and immobilized acetylcholinesterase inhibition in pesticide solution. Reprinted with permission from [40].

Chauhan and Pundir fabricated an organophosphorus (OP) mediator type biosensor based on covalently immobilizing the AChE purified from maize seedlings onto the composite of iron oxide nanoparticles and functionalized multiwall carbon nanotubes forming an AChE/Fe_3_O_4_/c-MWCNT/Au electrode (Figure 4) [40]. The synergistic action of Fe_3_O_4_NP and c-MWCNT showed excellent electrocatalytic activity at substantial low potential (+0.4 V). The biosensor exhibited good sensitivity (0.475 mA/μM), reusability (more than 50 times) and stability (two months) as compared to the unmodified one. A description of a novel glucose biosensor was proposed, based on the immobilization of glucose oxidase (GO_x_) with cross-linking in the matrix of biopolymer chitosan (CS) on a glassy carbon electrode (GCE) [41]. The electrode was modified with gold-platinum alloy nanoparticles (Au-PtNPs) through electrodeposition on multiwall carbon nanotubes (MWCNTs) in CS film (MWCNTs/CS). Au-PtNPs/CNTs had a better synergistic electrocatalytic effect on the reduction of hydrogen peroxide than did AuNPs/CNTs or PtNPs/CNTs at a low applied potential window. The biosensor exhibited excellent performances for glucose at a low applied potential (0.1 V) with a high sensitivity, a low detection limit of 8.53 μA mM^−1^ and 0.2 μM respectively. A wide linear range (0.001–7.0 mM), a fast response time (<5 s), good reproducibility, stability, and selectivity were also reported. The biosensor was applied in the determination of glucose in human blood and urine samples, and satisfactory results were achieved. The sensitivity of the nanocomposite was enhanced due to the excellent electrocatalytic activities of the carbon nanotubes. Ndlovu *et al.* demonstrated the electrochemical detection of *o*-nitrophenol on a poly(propylene)-gold nanocomposite modified glassy carbon electrode [8]. A reduction peak with enhanced current was observed and the reduction peak current was proportional to *o*-nitrophenol concentration in the range of 6.1 × 10^−7^ mol/L–6.25 × 10^−4^ mol/L. The detection limit was 4.5 × 10^−7^ mol/L. The sensor showed good reproducibility and stability.

A simple and sensitive electrochemical sensor for detection of nitrite was fabricated by construction of Au nanoparticles on the surface of choline chloride monolayer modified GCE [42]. The nano-Au/Ch film provided a porous structure with large effective surface area, which could act as electron transfer medium and promote charge transfer. The electrochemical sensor exhibited strong electrocatalytic activity towards the oxidation of nitrite. The designed sensor exhibited higher sensitivity of 0.354 μA/μM, a wider linear range of 4.0 × 10^−7^–7.5 × 10^−4^ M, a lower detection limit of 1.0 × 10^−7^ M and excellent stability. Moreover, this proposed method was applied to the determination of nitrite in natural water and sausage samples with satisfactory results. Direct electron transfer and multi-electrocatalysis was demonstrated using xanthine oxidase/laponite nanoparticles immobilized on glassy carbon electrode by Shan *et al.* [43]. Xanthine oxidase/laponite thin film modified electrode showed only one pair of well-defined and reversible cyclic voltammetric peaks attributed to XnOx-FAD cofactor at about −0.370 V *vs*. SCE (pH 5). The formal potential of XnOx-FAD/FADH_2_ couple varied linearly with the increase of pH in the range of 4.0–8.0 with a slope of −54.3 mV/pH. The immobilized XnOx retained its biological activity well and displayed an excellent electrocatalytic performance to both the oxidation of xanthine and the reduction of nitrate. The electrocatalytic response showed a linear dependence on the xanthine concentration ranging from 3.9 × 10^−8^–2.1 × 10^−5^ M with a detection limit of 1.0 × 10^−8^ M based on S/N = 3.

**Figure 5.**
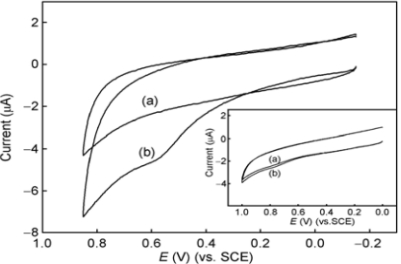
Cyclic voltammogram of the AChE/Au/Chi and AChE/Chi (inset) modified electrodes in pH 7.0 PBS in the absence (**a**) and presence (**b**) of 0.4 m M ATCL. Reproduced by permission from [45].

The use of ionic liquid and carbon nanomaterials at the electrode surface may increase the surface ionic and electrical conductivities thus enhance the sensitivity of the sensor. To complement this, Sun and co-workers [44] reported on a stable composite film composed of the ionomer Nafion, the ZnO nanoparticle and the protein hemoglobin which was cast on the surface of an ionic liquid modified carbon paste electrode (CILE) to establish a modified electrode denoted as Nafion/nano-ZnO/Hb/CILE. The electrochemical behaviours of hemoglobin (Hb) entrapped in the film were carefully investigated with cyclic voltammetry. A pair of well-defined and quasi-reversible redox voltammetric peaks for Hb Fe(III)/Fe(II) was obtained with the standard potential (*E^0^*) located at −0.344 V (*vs.* SCE) in phosphate buffer solution (PBS, pH 7.0), which was attributed to the direct electron transfer of Hb with electrode in the microenvironments of ZnO nanoparticle and ionic liquid 1-butyl-3-methylimidazolium hexafluorophosphate (BMIMPF_6_). The electrochemical parameters of Hb in the composite film were further carefully calculated with the results of the electron-transfer rate constant (*k_s_*) as 0.139 s^−1^, the charge transfer coefficient (*α*) as 0.413 and the number of electron transferred (*n*) as 0.95. The Hb modified electrode showed good electrocatalytic ability toward the reduction of trichloroacetic acid (TCA). YanRong *et al.* developed a simple method for immobilization of AChE on one-dimensional (ID) gold nanoparticles/chitosan colloid [45]. ID Au nanoparticles were prepared by electrodeposition in the pores of an alumina template. The immobilized AChE retained its catalytic activity. The work demonstrated that ID Au nanoparticles could serve as an ideal carrier for immobilization of AChE to fabricate the corresponding biosensor for organophosphorus insecticides as compared to bare glass carbon electrodes (Figure 5).

The biosensor exhibited satisfactory stability and reproducibility. Methamidophos could be determined in the range from 0.004–24 μg/mL with a detection limit of 0.001 μg/mL. Marinov *et al.* also reported on an amperometric acetylthiocholine sensor based on acetylcholinesterase immobilized on nanostructured polymer membrane containing gold nanoparticles [46]. Rakhi *et al.* developed a new amperometric biosensor, based on deposition of glucose oxidase (GOD) onto crystalline gold (Au) nanoparticle modified multiwalled carbon nanotube (MWNT) electrode [47]. Jha and Ramaprabhu also used gold nanoparticles dispersed on the outer surface of multiwalled carbon nanotubes (Au-MWNTs) as the electrode material, as it possesses high electron transfer rates and provides large immobilization sites for the bioenzymes, which combines with the high electrocatalytic activity of MWNTs for thiocholine oxidation at low potential [48]. The biosensor was found to be disposable and sensitive. The ability of the Au-MWNTs nanocomposite-based biosensor was applied to measure the concentration of paraoxon in the nanomolar range.

In another study involving nanoparticles, a self-assembly of gold nanoparticles (GNPs) over a self-assembled monolayer (SAM) of 3-aminopropyltriethoxysilane (APTES) on an indium-tin-oxide (ITO) coated glass plate was prepared as an enzyme immobilization matrix by Ahuja *et al.* [49]. The surface of the GNPs was modified with a mixed (1:9) SAM of 11-mercaptoundecanoic acid (MUA) and 3-mercapto-propionic acid (MPA). Covalent immobilization of uricase to the carboxyl groups of the mixed SAM of MUA/MPA through carbodiimide coupling reaction was achieved. The whole assembly was constructed on 1 cm^2^ area of ITO-glass plate and was tested as an amperometric biosensor for the detection of uric acid in aqueous solution. The biosensor assembly was characterized by atomic force microscopy (AFM) and electrochemical techniques. The AFM of the enzyme biosensor assembly revealed an asymmetrical sharp regular island-like structure with an average roughness parameter value of 2.81 nm. Chronoamperometric response was measured as a function of uric acid concentration in aqueous solution (pH 7.4), which exhibits a linear response over a concentration range of 0.07–0.63 mM with a sensitivity of 19.27 microA/mM and a response of 25 s with excellent reproducibility. The presence of interfering reagents such as ascorbic acid, urea and glucose were found to have no effect.

### 2.2. Sol-Gel Polymeric Materials

Recently, research is being focused on the use of composites based on sol-gel and other materials in the process of enzyme electrode fabrication. Sol-gel materials provide a versatile way for immobilization due to the presence of inorganic M–O or M–OH–M bridges forming a continuous network containing a liquid phase which can then be dried out to form a solid, porous polymeric matrix [1]. Salimi *et al.* fabricated a glucose biosensor by immobilizing glucose oxidase into a sol-gel composite at the surface of a basal plane pyrolytic graphite (bppg) electrode modified with multiwall carbon nanotube [50]. The carbon nanotubes offered excellent electrocatalytic activity toward reduction and oxidation of hydrogen peroxide liberated in the enzymatic reaction between glucose oxidase and glucose. The amperometric detection of glucose was carried out at 0.3 V, sensitivity of 196 nA/mM and detection limit of 50 μM (S/N = 3) were reported. The carbon nanotube sol-gel biocomposite glucose oxidase sensor showed excellent properties for sensitive determination of glucose with good reproducibility, remarkable stability, and rapid response and in comparison to bulk modified composite biosensors, the amounts of enzyme and carbon nanotube needed for electrode fabrication were dramatically decreased. Sun *et al*. reported on a room-temperature ionic liquid N-butylpyridinium hexafluorophosphate as a binder to construct an ionic liquid modified carbon paste electrode [51]. The modified electrode was characterized by scanning electron microscopy and electrochemical impedance spectroscopy. The ionic liquid carbon paste electrode (IL-CPE) showed enhanced electrochemical response and strong analytical activity towards the electrochemical oxidation of dopamine (DA). A pair of well-defined quasi-reversible redox peaks of DA appeared, with the redox peaks located at 215 mV, 151 mV for E_pa_ and E_pc_ respectively. The formal potential (E*^0^*) was calculated as 183 mV (*vs*. SCE) and the peak-to-peak separation was reported as 64 mV. The anodic peak currents increased linearly with the concentration of DA in the range 1.0 × 10^−6^–8.0 × 10^−4^ mol/L and detection limit was calculated as 7.0 × 10^−7^ mol/L. The interferences of foreign substances were investigated and the proposed method was successfully applied to the determination of DA injection samples. The IL-CPE designed was sensitive, selective and showed good ability to distinguish the coexisting ascorbic acid and uric acid. The electrochemical behaviours of rutin at the MWNTs-IL-Gel/glassy carbon electrode (GCE) were investigated by Liu *et al*. [52]. Good electrocatalysis behaviour towards the oxidation of rutin with enhancement of the redox peak current and decrease of the peak-to-peak separation was observed. The electrochemical parameters of rutin were calculated giving values of the charge-transfer coefficient (*α*) and the electrode reaction standard rate constant (*ks*) as 0.47 and 0.2 s^−1^, respectively. The oxidation peak currents of rutin in such a modified electrode increased linearly with the concentration of rutin in the range from 7.2 × 10^−8^–6.0 × 10^−6^ mol/L with a detection limit of 2.0 × 10^−8^ mol/L. These results suggest that the proposed electrode can be used for sensitive, simple and rapid determination of rutin.

**Figure 6.**
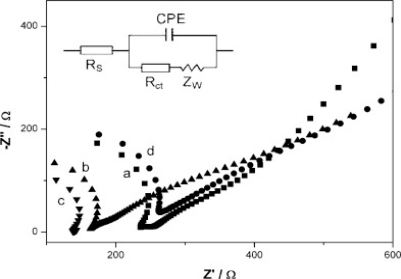
Nyquist plots for CP (**a**), MWCNT/CP (**b**), IL-MWCNT/CP (**c**) and IL-/CP (**d**) in the presence of 5 mM Fe(CN)_6_^3−^/5 mM Fe(CN)_6_^4−^ in the frequency range of 0.01 kHz–1 MHz with perturbation signal of 5 mV, at open potential (OPC) (insert-Randles circuit). Reproduced with permission from [57].

Developmen of an enzyme based biosenser for heavy metal ions determination was reported by IIangovan *et al*. [53]. A sol-gel-immobilized-urease conductometric biosensor on a thick film interdigitated electrode was used for heavy metal ions determination in synthetic effluents. The biosensor exhibited good response to changes in urea concentration within the range of 1 mM to 15 mM. Among the three metals studied, the amount of inhibition was found to have the following order cadmium, copper and then lead. In another study, an amperometric tyrosinase biosensor based on MWCNTs dispersed in mesoporous composite films of sol-gel-derived titania and Nafion was developed [54]. The enzyme tyrosinase was immobilized within the composite film. Phenolic compounds were determined by the direct reduction of biocatalytically liberated quinone species. This sensor exhibited remarkably fast response time, less than 3 s and a good performance in terms of the sensitivity (417 mA/M) and the detection limit (0.95 nM) due to the large pore size of the composite film. Zou *et al.* developed another amperometric glucose biosensor based on electrodeposition of platinum nanoparticles (PtNPs) onto MWCNTs and entrapping an enzyme in CS-SiO_2_ sol-gel [55]. The electrode showed an excellent electrocatalytic activity and high stability due to the synergistic action of Pt and MWCNTs and the biocompatibility of CS-SiO_2_ sol-gel. A wide linear range from 1 μM to 23 mM and a low detection limit of 1 μM was achieved for glucose determination. In another work, Sun *et al.* reported on two AChE immobilization methods on the surface of glassy carbon electrode (GCE) [56]. The immobilization methods employed a cross-linking method with glutaraldehyde as a cross-linking agent, bovine serum albumin (BSA) as a protectant, and sol-gel method with tetraethoxysilane (TEOS). AChE was immobilized on chitosan membranes by these two immobilization methods. Measuring the activity of the immobilized AChE by measuring current from the oxidation of thiocholine (TCh), produced by hydrolysis of the acetylthiocholine iodide (ATChI) substrate showed that the activity and stability of AChE employing sol-gel method with TEOS was higher than the cross-linking method with glutaraldehyde. Rotariu *et al*. illustrated that ionic liquid-multi-walled carbon nanotube (IL-MWCNT) composite gels present electrochemical properties toward thiocholine oxidation [57]. The modified IL-MWCNT electrodes allowed a stable, sensitive detection of thiocholine and the lowest oxidation potential (0–50 mV) compared to glassy carbon electrodes or chemically modified electrodes. Combining the high catalytic activity, fast electron transfer rate and large active area of the MWCNT with unique properties of ionic liquids, such as high ionic conductivity and ion exchange capacity, the IL-MWCNT gels proved to have an enhanced effect on thiocholine oxidation. The synergistic effect of the MWCNT and IL was demonstrated by using electrochemical techniques such as cyclic voltammetry and electrochemical impedance spectroscopy (EIS). The EIS in Figure 6 indicated that the formation of composite gel between the IL and MWCNT proved an enhanced electrocatalytic effect of the gels towards thiocholine oxidation.

Based on these favorable characteristics, a novel, sensitive, reusable and low potential acetylcholinesterase biosensor for chlorpyrifos based on 1-butyl-3-methylimidazolium terafluoroborate/multiwalled carbon nanotubes was further developed by Zamfir *et al.* [58]. The composite gel promoted electron transfer at low potential (+50 mV) and catalysed electrochemical oxidation of thiocholine with high sensitivity. AchE was immobilised in sol-gel matrix that provided a good support for enzyme without any inhibition effect from the ionic liquid. The detection limit of 4 nM for chlorpyrifos was obtained. Fast and efficient enzyme reactivation was obtained at low obidoxime concentration (0.1 mM) for the first time. Moreover, the developed biosensor exhibited a good stability and reproducibility and could be used for multiple determinations of pesticides with no loss of the enzyme activity.

In another study, the electrochemical behaviour of hydroquinone (HQ) was studied by cyclic voltammetry at a glassy carbon electrode (GCE) modified by a gel containing multi-walled carbon nanotubes (MWNTs) and room temperature ionic liquid (RTIL) of 1-butyl-3-methylimidazolium hexafluorophosphate (BMIMPF_6_) [59]. The HQ showed a pair of quasi-reversible redox peaks at the modified electrode. The cathodic peak current value (I_pc_) of HQ was 9.608 × 10^−4^ A, which is 43 times larger than the one at the bare GCE, and 11 times larger than that of I_pc_ at the MWNTs/GCE. Furthermore, the capabilities of electron transfer on these three electrodes were also investigated by electrochemical impedance spectroscopy (EIS), and similar conclusions were drawn from cyclic voltammetry. Recently, Skeika and coworkers presented a carbon ceramic electrode prepared by the sol-gel technique and modified with ferrocenecarboxylic acid [60]. Characterization of a carbon ceramic electrode modified with ferrocenecarboxylic acid (designated as CCE/Fc) was done by electrochemical techniques and its detection ability for dopamine. From cyclic voltammetric experiments, it could be observed that the CCE/Fc presented a redox pair at *E*_pa_ = 405 mV and *E*_pc_ = 335 mV (Δ*E* = 70 mV), related to the ferrocene/ferrocenium process. Studies showed a considerably increase in the redox currents at the same oxidation potential of ferrocene (*E*_pa_ = 414 mV *vs.* Ag/AgCl) in the presence of dopamine (DA), differently from those observed when using only the unmodified CCE, in which the anodic peak increase was considerably lower.

### 2.3. Conductive Polymeric Materials

**Figure 7.**
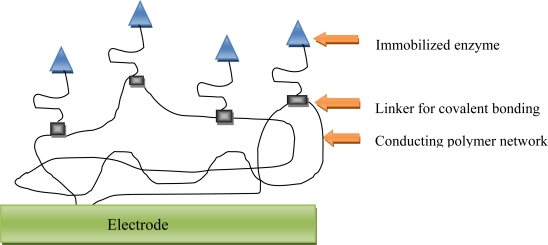
Schematic diagram of a conducting polymer/enzyme electrode with enzyme covalently bound to the polymer backbone.

During the recent years, electron-conducting polymers (Figure 7) have been investigated extensively. These can be grown electrochemically on the electrode and have been used in amperometric enzyme electrodes.

Stoces *et al.* reported on the dual functions of conducting polymers, of both binding the active enzyme and allowing for appropriate electron transfer reaction between enzyme and electrode through the network of the polymer [61]. A shuttle mechanism based on natural or artificial redox mediators is the basis of electron transfer [1]. To increase electron transfer, biocompatibility, porosity on glucose oxidase immobilized on reconstituted cellulose on glassy carbon electrode, the multiwalled carbon nanotubes were used to prepare the matrix with cellulose as investigated by Wu *et al.* [62]. These favorable results could mainly be attributed to two factors, firstly cellulose endowed with large amount of –OH groups, providing a biocompatible environment for encapsulation of glucose oxidase and the cellulose-MWCNT matrix possessing a porous structure, minimizing leaching and allowing a large amount of enzyme to be immobilized close to the electrode surface, where direct electron communication between active site of enzyme and electrode is enabled. Tumturk *et al.* used a natural polymer alginate, in the form of beads for immobilization of AChE [63]. The immobilized enzyme showed superior properties when compared to the free one in terms of reuse number, storage and thermal stability. Physicochemical properties of mediators such as large surface-to-volume ratio, high conductivity and good biocompatibility on excellent performance of biosensor were demonstrated using graphene and chitosan in forming a nanocomposite [64]. The glucose oxidase (GOD) immobilized on the electrode retained its bioactivity, exhibited a surface confined, reversible two-proton and two electron transfer reaction, and good stability, activity and a fast heterogeneous electron transfer rate with rate constant (*k_s_*) of 2.83 s^−1^. The biosensor exhibited a wider linearity range of 0.08 mM–12 mM glucose and a detection limit of 0.02 mM, and much higher sensitivity of 37.93 μA/mM as compared with other nanostructured supports. Zhiang *et al*. fabricated a novel polymerized film of acid chrome blue K (ACBK) on the surface of a glassy carbon electrode (GCE) by electropolymerization, and then the modified electrode was successfully used to simultaneously determine dopamine (DA), ascorbic acid (AA) and uric acid (UA). The poly-ACBK modified GCE exhibited excellent electrocatalytic activity towards the oxidations of DA, AA and UA in 0.05 mM phosphate buffer solution (pH 4.0) [65]. The detection limits (S/N = 3) were 0.5, 10.0 and 0.5 μM for DA, AA and UA, respectively. Considering the good selectively and sensitivity, the present method was applied to the determination of DA in dopamine hydrochloride injections, AA in vitamin C tablets and UA in urine samples.

Determination of Ag (1) by differential pulse voltammetry using a glassy carbon electrode modified with synthesized N-(2-aminoethyl)-4,4-bipyridine (ABP) was demonstrated [66]. ABP was covalently immobilized on GC electrode surface using 4-nitrobenzendiazonium (4-NBD) and glutaraldehyde (GA). The calibration curve was linear in the concentration range from 0.05 μM–1 μM Ag (1) with detection limit of 0.025 μM and RSD = 3.6%, for 0.4 μM Ag (1). The effect of several interferences was studied, and no effect on the determination of Ag (1) was observed. Oztekin *et al.* reported the electrochemical modification of glassy carbon (GC) electrode surface with the electro-polymerised form of 1,10-phenanthroline monohydrate (PMH) [67]. The characterization of polyphenanthroline modified electrode (PPMH/GC) was done by cyclic voltammetry and atomic force microscopy and the electroanalytical application suitable for the determination of Cd^2+^ ions was demonstrated by square wave voltammetry. The modified electrode was applied successfully to real samples.

Ivanov *et al.* reported on the effect of modification on the sensitivity of inhibitor determination for the first time [68]. Demonstration was done using cholinesterase sensors based on screen-printed electrodes modified with polyaniline, 7,7′,8,8′-tetracyanoquinodimethane (TCNQ), and Prussian blue. The slopes of the calibration curves obtained with the modified electrodes were increased two-fold and the detection limits for pesticides were reduced by factors of 1.6 to 1.8 in comparison with the use of unmodified transducers. In another study, thiocholine has also been determined using TCNQ as an electrochemical mediator. The measurement was carried out by differential pulse voltammetry technique reaching a detection limit equal to 2.5 × 10^−6^ mol/L. This sensor was also used as substrate for cholinesterase biosensor with applications in the food and environmental fields [69–71]. In another work by Florescu *et al.*, TCNQ was used to modify the screen-printed electrodes for determination of ionic heavy metals [72]. The sensibility of the sensor was 18.02 μA/mM in the presence of AChE compared with 0.53 μA/mM obtained for the TCNQ modified screen-printed electrode when the ions were detected directly. The above observations displayed that the TCNQ modified screen-printed electrodes can be used together with AChE enzyme to detect selectively and with high sensitivity the presence of heavy metal ions.

Sharma *et al.* reported on electrochemically prepared poly (aniline-*co*-fluoroaniline) films for immobilization of glucose oxidase using a physical adsorption technique [73]. A favorable result such as 15 days for the shelf life was obtained. Yin *et al.* developed a dopamine (DA) sensor using β-cyclodextrin-incorporated MWCNTs on a polyaniline (PANI) modified GCE [74]. The superior transducing property, rapid electron transfer capability of PANI and MWCNTs respectively, and the preconcentration by β-cyclodextrin showed the excellent sensitivity, selectivity, stability, and reproducibility in the determination of DA. Haccoun *et al*. reported on a reagentless lactate biosensor made of an electro-polymerized copolymer film made of poly (5-hydroxyl-1,4-naphthoquinone-*co*-5-hydroxyl-3-thioacetic acid-1,4-naphthoquinone) [75]. The electroactive specie quinone in the chain has been reported to work as an immobilized mediator and also providing low working potential when compared to other reported mediators in the literature. Polyaniline derivates such as poly (N-methylaniline [76,77], poly (N,N-dimethylamine) [78] have also been used frequently for biosensor applications. In another work, Du *et al.* designed a simple method to immobilize acetylcholinesterase (AChE) on polypyrrole (PPy) and polyaniline (PANI) copolymer doped with multi-walled carbon nanotubes (MWCNTs) [79]. The synthesized PANI-Ppy-MWCNTs copolymer presented a porous and homogeneous morphology which provided an ideal size to entrap enzyme molecules.

Recently, a conductive polyaniline (PANI) was electrochemically synthesized by controlled potential cycling as a film at the surface of a carbon paste electrode making a carbon paste (CP) biosensor. The aniline, as part of the polymer backbone, has the dual function, providing suitable conditions for the effective, one step enzyme immobilization (*i.e.*, without use of cross-linking agent) and, at the same time, acting as a mediator [61]. The immobilization of acetylcholinesterase on platinum microelectrodes modified with p-nitrobenzenediazonium was optimized [80]. The use of microelectrodes improved the detection limit of ethylparaoxon measurements to 20 nM compared to 100 nM in case of screen printed-printed electrodes based on the same method of immobilization.

In order to evaluate hexacyanoferrates as electrochemical mediators for thiocholine detection, Arduini *et al.* chose cobalt hexacyanoferrate (CoHCF) as modifier of the screen-printed electrodes [7]. The transfer coefficient (*α*) and the apparent charge transfer rate constant (*k_s_*) for electron transfer between the electrode and CoHCF layer was calculated. The CoHCF-SPE was used in amperometric batch conditions at +0.5 V *vs*. Ag/AgCl showing for thiocholine detection a good linear range (5 × 10^−7^–1 × 10^−5^ mol/L) with a low detection limit and high sensitivity equal to 5 × 10^−7^ mol/L and 435 mA mol^−1^ L cm^−2^, respectively. The good values of sensitivity and detection limit obtained displayed a good analytical performance when compared to other mediators adopted for thiocholine measurement such as cobalt phthalocyanine [81,82] and Prussian blue [83]. Electrochemical behaviour of cerium hexacyanoferrate (CeHCF) incorporated on multi-walled carbon nanotubes (MWNTs) modified GC electrode was investigated by Fang *et al.* [13]. The CeHCF/MWNT/GC electrode showed potent electrocatalytic activity toward the electrochemical oxidation of tryptophan in phosphate buffer solution (pH 7.0) with a diminution of the overpotential of 240 mV. The anodic peak currents increased linearly with the concentration of tryptophan in the range of 2.0 × 10^−7^ to 1.0 × 10^−4^ M with a detection limit of 2.0 × 10^−8^ M (S/N = 3).

Chaubey *et al.* described the immobilization of lactate dehydrogenase (LDH) on electrochemically polymerized polypyrrole-polyvinyl-sulphonate (PPY-PVS) composite films via a cross-linking technique using glutaraldehyde [84]. The characterization of the composite film was carried out using FTIR and cyclic voltammetry. The PPY-PVS-LDH electrodes were shown to have a detection limit of 1 × 10^−4^ M, response time of about 40 s, and a shelf-life of about 2 weeks. Lactate estimation from 0.5 to 6 mM was determined using this biosensor. Asberg and Inganas cross-linked horseradish peroxidase in highly conducting poly (3,4-ethylene dioxythiophene) PEDOT/(polystyrene sulphonate) (PSS) poly-4-vinylpyridine for estimation of hydrogen peroxide in the concentration range of 0–30 μm [85]. Kok *et al.* co-immobilized acetylcholinesterase and choline oxidase on membranes [86]. Immobilization of the enzymes was achieved either by entrapment or surface attachment via a hybrid immobilization method. Aldicarb detection studies showed that a linear working range of 10–500 and 10–250 ppb aldicard could be achieved by the entrapped and surface immobilized enzymes, respectively. Enzymes immobilized on membrane surfaces responded to aldicard presence more quickly than entrapped enzymes.

Bontidean *et al.* described a novel capacitance biosensor based on synthetic phytochelatins for sensitive detection of heavy metals [87]. Synthetic phytochelatin (Glu-Cys)_20_Gly(EC20) fused to the maltose binding domain protein was expressed in *Escherichia coli* and purified for construction of the biosensor. The biosensor was able to detect Hg^2+^, Cd^2+^, Pb^2+^, Cu^2+^ and Zn^2+^ ions in the concentration range of 100 fM–10 mM, and the order of sensitivity was S_Zn_ > S_Cu_ > S_Hg_ >> S_Cd_ ≈ S_Pb_. Regeneration of the sensor was done using EDTA and storage stability of the biosensor was 15 days. Malitesta and Guascito employed an inhibition scheme for detecting Hg^2+^ based on glucose oxidase immobilized in poly-*o*-phenylenediamine [88]. The investigated enzyme inhibition appeared reversible and mixed, in agreement with data for the enzyme in solution. The most interesting characteristics were a low response time (<2 min) and rapid recovery of the response of EDTA. Wang *et al.* developed a novel electrochemical sensor based on molecularly imprinted polymer film for aspirin detection [89]. The sensitive film was prepared by co-polymerization of *p*-aminothiophenol (p-ATP) and HAuCl_4_ on the Au electrode surface. First, p-ATP was self-assembled on the Au electrode surface by the formation of Au-S bonds. Then, the acetylsalicylic acid (ASA) template was assembled onto the monolayer of p-ATP through the hydrogen-bonding interaction between amino group (p-ATP) and oxygen (ASA). Finally, a conductive hybrid membrane was fabricated at the surface of Au electrode by the co-polymerization in the mixing solution containing additional p-ATP, HAuCl4 and ASA template. The ASA was spontaneously imprinted into the poly-aminothiophenol gold nanoparticles (PATP-AuNPs) complex film. The amount of imprinted sites at the PATP-AuNPs film increased due to the additional replenishment of ASA templates. The increase of imprinted sites and doped gold nanoparticles, led to the sensitivity of the molecular imprinted polymer (MIP) electrode gradually increasing. Favorable characteristics were obtained, the linear relationships between current and logarithmic concentration were obtained in the range from 1 nmol/L–0.1 μmol/L and 0.7 μmol/L–0.1 mmol/L. The detection limit of 0.3 nmol/L was achieved.

### 2.4. Functional Polymeric Materials

**Figure 8.**
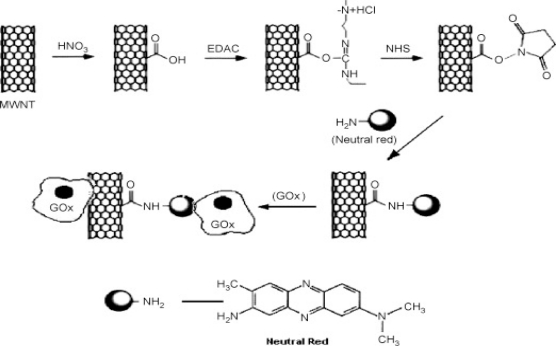
Schematic representation of the covalent attachment of neutral red with MWNTs and physical adsorption of GOx over the functionalized MWNTs. Reprinted with permission from [91].

These are polymeric materials which possess different functional groups, for example, thiols, amines, carboxylic acids and others before modification or after modification of the surface using different oxidizing agents. Functional polymers were used to construct a novel biosensor for glucose measurements [90]. The physical and chemical functionality of hydrophobic polydimethylsiloxane (PDMS) and hydrophilic 2-methacryloyloxyethyl phosphorylcholine (MPC) copolymerized with dodecylmethacrylate (DMA) were utilised. Jeykumari and Narayanan developed a nanobiocomposite of MWCNTs functionalized with Neutral Red (NR), GO_x_ as the biocatalyst and Nafion (Nf) as the binder to get a robust modification of the electrode surface (Figure 8) [91].

The bio-catalytic activity of this MWCNT-NR-GO_x_-Nf nanobiocomposite modified electrode showed good characteristics such as low potential detection of glucose with a large determination range from 1 × 10^−8^–1 × 10^−3^ M with detection limit of 3 × 10^−9^ M glucose, a short response time, good stability and anti-interference ability. The application of carbon modified by the reduction of aromatic diazonium derivatives was first demonstrated for the electrochemical analysis of heavy metals by Fan *et al*. [92]. The anodic peak currents of cadmium and lead at the benzoic acid-modified glassy carbon electrode (BA/GCE) were 7.2 and 6 times of that at the bare glassy carbon electrode. A linear response was observed for Pb^2+^ and Cd^2+^ in the range of 0.5–50 μg/L. The detection limits were 0.2 μg/L and 0.13 μg/L for Pb^2+^ and Cd^2+^ respectively. The BA/GCE showed stability and improved sensitivity. The sensor was successfully applied to the determination of Pb^2+^ and Cd^2+^ in sewerage samples.

Le *et al.* chose mesoporous carbon (MC) and carbon black (CB) as an anodic layer for developing a novel organophosphorus hydrolase (OPH) biosensor for detecting organophosphate chemicals [93]. The sensitivity of the sensor towards *p*-nitrophenol (PNP) was greatly improved. The MC/CB/glass carbon (GC) layer exhibited an enhanced amperometric response relative to a carbon nanotube (CNT)-modified electrode because it promoted electron transfer of edge-plane-like defective sites and high surface area of the MC resulted in increased sensitivity and allowed for nanomolar-range detection of the analyte paraoxon. The biosensor had a detection limit of 0.12 μM (36 ppb) and a sensitivity of 198 nA/μM for paraoxon. The use of a glassy carbon electrode (GCE) modified by activated carbon (AC) mediating the redox of mercuric chloride (HgCl_2_) in 0.1 M aqueous solution of potassium chloride (KCl) supporting electrolyte was demonstrated [94]. An oxidation and two reduction peaks of Hg^2+^ appeared at +200, +680 and +100 mV respectively, *versus* Ag/AgCl during electrochemical studies. The redox current of Hg^2+^ was enhanced by two folds at AC modified GCE and about five folds in acidic media. The oxidation peak of Hg^2+^ was shifted to lower potential by approximately 5 mV and for the reduction peak was shifted to 0 mV in acidic solution at AC/GCE. The sensitivity under condition of cyclic voltammetry was significantly dependent on pH, concentration of AA and temperature. Interference with Hg^2+^ was observed in different metal ions, such as Ca^2+^, Cu^2+^, Ni^2+^, Mn^2+^ and Cd^2+^. The current enhancement appeared and caused further increase in the reduction peaks of Hg^2+^, in contrast the oxidation current decreased when increasing the concentration of the interference metals.

Rahimi *et al.* reported on a nanocomposite containing amine functionalized multiwall carbon nanotubes and 1-butyl-3-methylimidazolium tetrafluoroborate, a room temperature ionic liquid [95]. The composite was applied to glucose oxidase (GO_x_) immobilization on glassy carbon electrode. The proposed nanocomposite provided a favorable microenvironment to preserve the bioactivity of GO_x_ and effectively facilitated the direct electron transfer to the electrode as evidenced by increase in ip_c_ and ip_a_ from cyclic voltammetry as compared to bare glassy carbon electrode. This brought about a remarkable improvement in sensitivity and response time of the glucose biosensor with values of 1,277 μA/mM cm^2^ and 6 s respectively. The biosensor provided a linear dynamic response between 0.1 and 43 μM with a very low detection of 63 nM. The values for the apparent Michaelis-Menten constant and maximum current were obtained as 18 μM and 2.7 μA, respectively. A glassy carbon electrode modified with functionalized multiwalled carbon nanotubes (CNTs) immobilized by 1-ethyl-3-(3-dimethylaminopropyl) carbodiimide/N-hydroxysuccinimide (EDC/NHS) in a dihexadecylphosphate film was prepared and characterized by cyclic voltammetry [96]. It was used as a support for FAD or glucose oxidase (GO_x_) immobilization with EDC/NHS crosslinking agents. Characterisation of GO_x_ immobilized onto the surface of CNTs showed a pair of well-defined redox peaks, which correspond to the direct electron transfer of GO_x_, with a formal potential of −418 mV *vs*. Ag/AgCl (3 M KCl) in 0.1 M phosphate buffer solution (pH 7.0). An electron transfer rate constant of 1.69 s^−1^ was reported. The half wave potential was shown to depend on pH indicating that the direct electron transfer reaction of GO_x_ involves a two-electron, two-proton transfer. The determination of glucose was carried out by square wave voltammetry and the developed biosensor showed good reproducibility and stability. Ragupathy *et al.* reported on the synergistic contributions of thiol multiwall carbon nanotubes (MWNTSH) and gold nanoparticles in a chitosan-ionic liquid matrix towards improved performance for a glucose sensor [97]. Direct electron transfer between glucose oxidase (GO_x_) and electrode was achieved. Scanning electron microscopy and atomic force microscopy images of GO_x_/Au/CS-IL-MWNT (SH) revealed that MWNTs and AuNPs are dispersed in CS-IL matrix. Cyclic voltammetry, impedance spectroscopy and chronoamperometry were used to evaluate the performance of biosensor. The GO_x_/Au/CS-IL-MWNT (SH) biosensor exhibited a linear current response to glucose concentration (1–10 mM) at a low potential of 0.10 V and precludes interferences from uric acid and ascorbic acid. The GO_x_/Au/CS-IL-MWNT (SH) biosensor was found to have superior performances over GO_x_/CS-IL-MWNT (SH). In order to have a common framework for analysis of biosensors discussed above an overview of some enzymatic and enzyme-free polymeric material based biosensors along with their important parameters is presented in Table 1.

### 2.5. Biomaterials

The removal of heavy metals from aqueous media by using biosorption biomaterials including bacteria [98], yeast [99–101] mosses [102], rice husk [103], modified corn cobs [104], tassel [105] have been widely reported for a long time. Recently, the biosorption process has been combined with electrochemical methods for sensitive determination of heavy metals. The use of biomaterial as modifying agents for the preparation of sensors has attracted much interest.

These biomaterials have been shown to contain different functional groups and binding sites for heavy metals as evidenced by microscopic and spectroscopic techniques [105–107]. Carbon paste electrodes have been used widely as suitable matrix for the preparation of modified electrodes due to simple preparation, renewability, and compatibility with various types of modifiers [15]. Electrical detections of metal ions have been studied using plant /microbial biomass modified electrodes and considerable improvements in electrochemical behaviour of metals have been displayed. The use of carbon electrodes chemically modified by algae [108], lichen [109], moss [110], chicken feathers [111,112], orange essential oil [113], pineapple [114], plant refuses such as apple, potato and cabbage peelings [115] for the biosensing of heavy metals such as Pb^2+^, Hg^2+^, Cu^2+^, Co^2+^, Cd^2+^, Zn^2+^ in aqueous solutions by cyclic voltammetry and differential pulse polarography have been reported.

**Figure 9.**
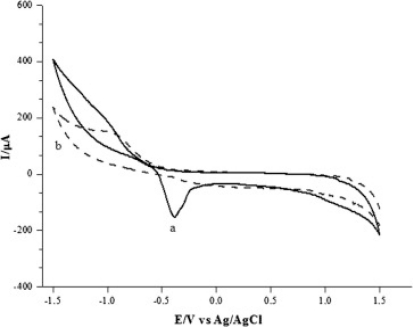
Cyclic voltammograms of *R. arrhizus* modified CPE (**a**) and unmodified CPE (**b**) (concentration of Pb(II): 1.0 × 10^−4^ M, detection: cyclic voltammetry in 0.01 M Tris-HCl with 50 mV/s scan rate, preconcentration time: 15 min). Reprinted with permission from [120].

In another study, a voltammetric detection of Lead(II) ions at a carbon paste electrode modified with banana tissues was described by Mojica *et al*. [116]. A limit of detection of 0.1 mg/L and a linear range from 1–20 mg/L was reported. Alpat *et al.* fabricated a novel biosensor based on carbon paste electrode consisting of whole cells of *Circinella sp* [117]. Cu^2+^ was preconcentrated on the electrode and then cathodically detected with the reduction of Cu^2+^, The developed biosensor exhibited an excellent current response to Cu^2+^ over a linear range from 5.0 × 10^−7^–1.0 × 10^−5^ M with a detection limit of 5.4 × 10^−8^ M. The microbial biosensor had good sensitivity and reproducibility (R.S.D. 4.3%, n = 6). The biosensor was applied to voltammetric determination of Cu^2+^ in real samples and the results were consistent with the one from the AAS method. A highly sensitive and fast responding electrochemical sensor was also prepared for Cu(II) by making a paste of *Rhodotorulla mucilaginosa*, graphite powder and paraffin liquid by Yüce *et al*. [118]. The developed biosensor was characterized by cyclic voltammetry, differential pulse stripping voltammetry, and SEM-EDAX analysis. The biosensor had a linear range of between 1.0 × 10^−7^ and 1.0 × 10^−5^ M Cu(II). A new biosensor system was reported for the amperometric detection of Cu(II) using recombinant *S.cerevisiae* strains as the biomaterial. The biosensor had a linear range between 5.0 × 10^−4^ and 2.10 × 10^−3^ M Cu(II) [119]. The use of *Rhodotorulla mucilaginosa* as biomaterial compared to *S.cerevisiae* provided more sensitivity and a wider range for determination of copper ions. Yüce *et al.* described the use of *Rhizopus arrhizus* as a sensor modifying component for the determination of Pb(II) in aqueous media by voltammetry [120]. The advantage of modifying the electrode in biosensing was illustrated by cyclic voltammetry (Figure 9) which shows a well-defined cathodic peak corresponding to reduction of surface bonded Pb(II) in a potential range of −0.6 to −0.4 V. A carbon paste electrode modified with dried nonliving biomaterial was used due to low cost of fabrication, low background current and renewability. The preconcentrated ions at open circuit were reduced by using differential pulse stripping voltammetry technique. The linear range of the biosensor was found to be within 1.0 × 10^−7^–1.25 × 10^−5^ M, with detection limit of 0.5 × 10^−8^ M. The effect of interference from other heavy metals on the microbial biosensor was investigated during the study. Energy dispersive X-ray was used to show the specific effect of the fungal biomass on the Pb(II) determination.

Yüce *et al.* described an advanced investigation on a new voltammetric algal sensor based on *Phormidium sp*. for Pb(II) determination from aqueous solution [121]. The linear range was comparable to the one obtained in the previous study, 5.0 × 10^−8^ M–2.0 × 10^−5^ M. The detection limit was 2.5 × 10^−8^ M. Possible functional such as carboxyl, sulphoxide and alcoholic groups were involved in the accumulation of Pb^2+^ as evidenced from FT-IR analysis spectrum.

Generally, the use of some biomaterials for the detection of different heavy metals in aqueous solutions through electrochemical means has been demonstrated. However, some of the biomaterials lack the very high sensitivity and very low detection limit required. The limitations of some of the biomaterials, for example micro-organisms, require special culture conditions in laboratories and skilled personnel. Consequently, search for other low cost biomaterials such as maize tassel which has been shown to adsorb a range of trace metals from aqueous solution has gather momentum [105,122–125]. Maize tassel is obtained from the maize plant which is a staple food in most countries, hence is locally available in large quantities and easily regenerated or discarded, thereafter with minimal environmental impacts. The characterization of maize tassel was reported [126] and was shown to have mesoporous morphology according to Brunaer-Emmett-Teller (BET) result. The High-resolution scanning electron microscopy (HRSEM) shows that the material is flattish with no porosity. The FT-IR showed functional groups such as ─NH_2_, ─C═O and ─COOH. The presence of these groups shows that maize tassel may have the ability to conduct electrically and mediation of electrons between the electrode and the immobilized enzyme might occur. Some of the biomaterials used for the detection of environmental pollutants are compared with other kinds of polymeric based materials and the results are listed in Table 2. It can be seen that these cheap, easily available materials can provide a comparable linear range and detection limit by cyclic voltammetry with a simple electrode procedure hence future use of biomass material in electrode modification is encouraged.

## 3. Conclusions and Future Perspectives

Proper electrode fabrication using different materials for efficient electron transport is very important. Recent advances in the use of polymeric materials such as nanomaterials, sol-gel materials, conducting polymers, functional polymers, and biomaterials have been discussed. Analytical sensing at electrodes modified with polymeric materials results in low detection limits, high sensitivities, lower applied potential, reduction of background, efficient electron transfer and easier immobilization of enzymes on electrodes such that sensing and biosensing of environmental pollutants is made easier. However, there are a number of challenges to be addressed in order to fulfill the applications of polymeric based polymers. The commercial production of pure and defect-free polymers is difficult and costly. For example, processing of carbon nanotubes is still not fully controlled, aggregations of tubes are not prevented, and are not uniformly obtainable; tube lengths are not reproducible, insoluble in most solvents and the health risks are clearly understood. On the other hand, long laboratory synthetic pathways and costs are involved in the production of the functional, sol-gel and conducting based polymeric materials. The aforementioned disadvantages of the above polymeric materials call for search for low cost biomaterials as alternative for the development of novel electrochemical sensors and biosensors. The banana, pineapple and feathers modified carbon paste electrodes exhibited a high sensitivity and a low detection limit, hence other low cost agro-based materials such as maize tassel, which has shown potential for trace metal removal, could also be used as electron mediators and immobilization matrix in biosensor development design. This material can be blended with sol-gel or nanomaterials to enhance the physicochemical properties so that efficient electron transfer between the electrode and enzyme is improved. It is believed that the merits of biomaterial based sensors will bring dramatic changes in future sensor technology.

## Figures and Tables

**Table 1.** Overview of some enzymatic and enzyme-free polymeric material based biosensors, along with their important parameters.

**Biosensor**	**Substrate**	**Sensitivity**	**Detection limit**	**Michaelis-Menten constant**	**Linear range**	**Oxidation and Reduction potential range**	**Stability**	**Refs**
**CoHCF-SPE**	Thiocholine	435 mA mol^−1^ L cm^−2^	5 × 10^−7^ M	-	5 × 10^−7^–1 × 10^−5^ M	−50 to 1,200 V	1 month	[7]
**GO_x_-MWCNT/GCE**	Glucose	11.3 μA/mM cm^2^	30 μM	-	0.5–40 nM	0.0 to +0.6 V	80% after 1 month	[19]
**CNT/OPH**	Paraxon	25 nA/μM	0.15 μM	-	-	−0.2 to +1.2 V	-	[20]
	Methyl-parathion	25 nA/μM	0.8 μM					
**Hb/Fe_3_O_4_/chitosan**	Hydrogen peroxide	-	4.0 × 10^−6^ M	0.29 mM	5.0 × 10^−5^–1.8 × 10^−3^ M 1.8 × 10^−3^–6.8 × 10^−3^ M	−0.4 to +0.6 V	89.5% after 30 days	[38]
**AChE/Fe_3_O_4_/c-MWCNT/Au**	Malathion	0.475 mA/μM	0.1 nM	-	0.1–40 nM	−0.5 to +1.0 V	2 months	[40]
	Chlorpyrifos		0.1 nM		0.1–50 Nm			
	Monocrotophos		1 nM		1–50 Nm			
	Endosulfan		10 nM		10–100 nM			
**GO_X_/Au-PtNPs/CNTs/GCE**	Glucose in human urine	8.53 μA/ mM	0.2 μM	-	0.001–7.0 mM	−0.5 to +0.5 V	90% after 35 days	[41]
**GO_X_/Bppg/MWCNT**	Glucose	196 nA/mM	50 μM	-	0.2–20 mM	−0.4 to +1.1 V	3 weeks	[50]
**AchE/[BMIM][BF_4_]/MWCNT**	Chlorpyrifos		4 nM	-	10^−8^–10^−6^ M	−400 to +800 mV	95% after 1 week	[58]
**AChE/MWCNT-C/GCE**	Acetylthiocholine	-	0.10 μmol/L	132 μmol/L	2.0–400 μmol/L	0.1 to 1.0 V	70% after 30 days	[30]
**PPY-PVS-LDH**	Lactate	-	1 × 10^−4^ M	9.4 mM	0.5 to 6 mM	-	2 weeks	[84]
**MWCNT-NR-GO_x_-Nf**	Glucose	-	3 × 10^−9^ M	-	1 × 10^−8^–1 × 10^−3^ M	−0.9 to −0.10 V	3 months	[91]

GO_x_, glucose oxidase; MWCNT, multiwalled carbon nanotube; GCE, glassy carbon electrode; CNT, carbon nanotube; OPH, organophosphorus hydrolase; Hb, haemoglobin; AChE, acetylcholinesterase; Fe_3_O_4_, iron oxide; PtNPs, platinum nanoparticles; [BMIM][BF_4_], 1-butyl-3-methylimidazolium terafluoroborate: Bppg, basal plane pyrolytic graphite: Au, gold; SPE, screen printed electrode; CoHCF, cobalt hexacyanoferrate;LDH, lactate dehydrogenase; PPY-PVS, polypyrrole-polyvinyl-sulphonate, NR, Neutral Red, Nf, Nafion; V, *vs.* saturated calomel electrode (SCE); V, *vs.* Ag/AgCl.

**Table 2.** Comparison of biomaterials based electrochemical sensors with enzyme-free polymeric based electrochemical sensors for detection of heavy metal ions and organics.

**Electrodes**	**Analyte**	**Linear range**	**Detection limit**	**Refs**
**F/CPE**	Pb(II)	1–10 mg/L	0.59 mg/L	[111]
**F/CPE**	Pb(II)	0.5–5 mg/L	0.121 mg/L	[112]
**B/CPE**	Pb(II)	-	0.10 mg/L	[116]
**MWCNT/ C/GCE**	tryptophan	2.0 × 10^−7^–1.0 × 10^−4^ M	2.0 × 10^−8^ M	[13]
**N/MWCNT/GCE**	Pb(II)	-	5.0 × 10^−9^ M	[32]
**S/N/MWCNT**	phenol	-	0.95 nm	[54]
**CNT/Au-Pt/GCE**	H_2_O_2_	-	0.2 μM	[29]
**R.a/CPE**	Pb(II)	1.0 × 10^−7^–1.25 ×10^−5^ M	0.5 × 10^−8^ M	[120]
**C.sp/CPE**	Cu(II)	5.0 × 10^−7^–1.0 × 10^−5^ M	5.4 × 10^−8^ M	[117]
**Algal/CPE**	Pb(II)	5.0 × 10^−8^–2.0 ×10^−5^ M	2.5 × 10 ^−8^ M	[121]

F, feathers; CPE, carbon paste electrode; B, banana; MWCNT, multiwalled carbon nanotube; C, cerium hexacyanoferrate; GCE, glassy carbon electrode; N, nafion; S, Sol gel; CNT, carbon nanotube; Au, gold; Pt, platinum; R.a, *Rhizopus arrhizus;* C.sp, *Circinella sp*.
